# Freeze-Drying Enhances Functional Properties and Sensory Acceptability of Cranberry Bean (*Phaseolus vulgaris* L.) Flour in Gluten-Free Cupcakes

**DOI:** 10.3390/foods15183282

**Published:** 2026-09-17

**Authors:** Nour Elhouda Bouhlel, Sana Azeiez, Ahmed Gomaa, Lazhar Zourgui, Krista A. Power, Wejdene Mansour, Riadh Hammami

**Affiliations:** 1 School of Nutrition Sciences, Faculty of Health Sciences, University of Ottawa, Ottawa, ON K1N 6N5, Canada; nbouh067@uottawa.ca (N.E.B.); agomaa@uottawa.ca (A.G.); krista.power@uottawa.ca (K.A.P.); 2 Research Laboratory “Metabolic Biophysics and Applied Pharmacology” LR12ES02, Faculty of Medicine of Sousse, University of Sousse, Sousse 4002, Tunisia; sana.azaiez@famso.u-sousse.tn; 3 Research Laboratory BMA “Biodiversity, Molecules, Application”, Higher Institute of Applied Biology Medenine, Gabes University, Gabes 6029, Tunisia; lazhar.zourgui@gmail.com; 4 Department of Biochemistry, Microbiology and Immunology, Faculty of Medicine, University of Ottawa, Ottawa, ON K1H 8M5, Canada

**Keywords:** cranberry bean flour, freeze-drying, oven-drying, gluten-free cupcakes, functional properties, texture profile analysis, sensory acceptability

## Abstract

While legume flours can improve nutritional quality of gluten-free baked products, the influence of drying methods on precooked cranberry bean (*Phaseolus vulgaris* L.) flour and its baking performance remain underexplored. This study compared oven-drying (OD) and freeze-drying (FD) effects on the physicochemical and nutritional properties of cranberry bean flour (CBF) and evaluated their impact on cupcake quality. Functional properties, proximate composition, mineral content, and total phenolic compounds of CBF, along with color, moisture content, texture, storage behavior, and sensory attributes of cupcakes were assessed. FD significantly increased water absorption capacity (3.44 ± 0.16 vs. 2.80 ± 0.02 g/g for OD; *p* = 0.02), oil absorption capacity (2.10 ± 0.13 vs. 0.98 ± 0.03 g/g; *p* = 0.003), and total phenolic content (1.23 ± 0.006 vs. 1.18 ± 0.008 mg GAE/g; *p* < 0.001), while producing a finer particle size (445 ± 86 vs. 1187 ± 210 nm; *p* = 0.02). OD retained significantly higher potassium, phosphorus, iron and zinc concentrations, whereas FD yielded a higher caloric value (401 ± 1.6 vs. 392 ± 0.9 Cal/100 g; *p* = 0.02). In cupcakes, FD flour produced significantly lighter crumb color than OD. Both bean-based cupcakes, prepared with an identical formulation, exhibited high moisture content (37.7–40.1%). Springiness was higher in OD cupcakes (14.5 ± 2.7 vs. 8.9 ± 1.7 mm; *p* = 0.023), while all other textural parameters were comparable. After 7-day storage, bean formulations retained over 27% moisture and showed comparable storage behavior. Sensory evaluation (*n* = 45) revealed that FD cupcakes had significantly lower bean flavor intensity (*p* = 0.017) and higher aroma liking (*p* = 0.0002) than OD cupcakes, suggesting improved consumer acceptability. Overall, freeze-dried CBF is a promising functional ingredient for developing nutritionally enhanced gluten-free cupcakes with favorable texture and sensory characteristics.

## 1. Introduction

The global market for gluten-free products has expanded considerably, driven by the increasing prevalence of celiac disease, affecting approximately 1% of the global population, and non-celiac gluten sensitivity, which affects about 6%, as well as by growing consumer demand for gluten-free dietary options [1,2]. As lifelong gluten exclusion remains the only effective treatment strategy for gluten-related disorders [3], increasing demand for nutritious and convenient gluten-free foods has intensified both industrial and research efforts toward the development of unconventional flour alternatives to replace wheat flour in baked products [4]. However, conventional gluten-free products, often formulated with refined starches such as rice flour, remain nutritionally limited, being low in protein, dietary fiber, vitamins, and minerals [5,6,7], and also suffer from critical structural and quality deficiencies, including poor dough cohesion and elasticity, brittle crumb texture, and rapid staling [3,8,9].

Legume flours have attracted increasing research interest as functional ingredients in gluten-free formulations, offering an affordable and nutrient-rich source for quality improvement through protein–starch interactions that enhance batter viscoelasticity and crumb structure [7,10,11]. Red kidney bean flour has been reported to improve viscoelastic properties and reduce firmness in gluten-free cupcakes [12,13], while kidney bean protein isolates have been shown to enhance the texture of starch-based gluten-free muffins [12]. Kidney bean pod powder has been evaluated in the optimization of gluten-free cake formulations [14], and legume protein isolates from kidney bean, field pea, and amaranth have demonstrated improvements in the structural and textural characteristics of gluten-free muffins [15]. Germinated colored bean flours have further been shown to confer favorable technological, bioactive, and sensory qualities in rice flour-based gluten-free muffin formulations [16]. Among legume varieties, cranberry bean (*Phaseolus vulgaris* L.) stands out for its exceptional nutritional profile, providing approximately 22 g of protein per 100 g dry weight, along with dietary fiber, essential minerals (iron, calcium, magnesium), and bioactive phenolic compounds with demonstrated antioxidant activity [17,18,19]. Despite these nutritional attributes and the documented benefits of other legume flours in gluten-free baking, cranberry bean flour has been very limitedly investigated as an ingredient in gluten-free baked products, representing a significant gap in the literature. Furthermore, while consumer acceptability remains a key challenge for legume-based products due to characteristic “beany” flavors and aromas, processing methods that minimize these sensory attributes while preserving nutritional quality have not been systematically evaluated.

The drying method used to process legume flours significantly influences their nutritional and functional properties, with downstream consequences for baking performance. Oven-drying (OD) is an accessible and cost-effective approach widely used in food processing, but the application of heat can induce protein denaturation, starch gelatinization, and degradation of heat-labile bioactive compounds, including phenolics and vitamins [20,21,22,23,24,25]. In contrast, freeze-drying (FD) operates at sub-zero temperatures under vacuum, preserving the native molecular structure of proteins and bioactive compounds while producing a porous, highly hygroscopic flour with superior rehydration properties [26,27]. While the effects of drying methods on flour properties have been reported for various plant materials [21,22,25,26,28], direct comparisons between OD and FD for precooked legume flours and their subsequent impact on gluten-free baking performance remain limited in the literature. Understanding how drying methods affect both flour functionality and final product quality is essential for optimizing legume flour processing and maximizing their potential in gluten-free product development.

Despite the documented effects of drying methods on flour properties and the growing interest in legume-based gluten-free products, no studies have compared oven-drying and freeze-drying for precooked cranberry bean flour and evaluated their impact on gluten-free baking performance and sensory acceptability. This study aimed to: (i) compare the effects of oven-drying and freeze-drying on the physicochemical properties of precooked cranberry bean flour, including functional properties, proximate composition, mineral content, and total phenolic compounds; and (ii) evaluate the impact of these drying methods on the quality characteristics of gluten-free cupcakes, including color, moisture content, texture profile, storage behavior, and sensory attributes.

## 2. Materials and Methods

### 2.1. Raw Material

**Figure 1:**
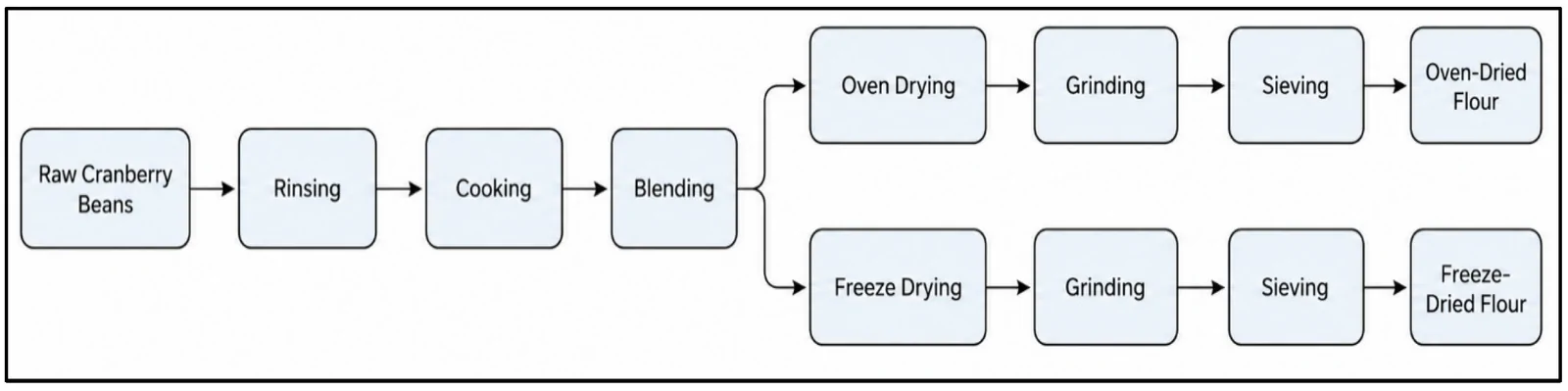
Process flow diagram for cranberry bean flour production using oven-drying and freeze-drying methods.

Cranberry beans (*Phaseolus vulgaris* L.) were purchased from a local farmer (Sousse, Tunisia). Beans were randomly divided into two batches. The first batch was transported to the Bio-Gatrana facility (Sidi Bouzid, Tunisia); in collaboration with this partner, the cooking and oven-drying parameters were optimized following the methodology described by Rodrigue [29]. The second batch was transferred to the food-grade laboratory at the Faculty of Health Science, University of Ottawa (Ottawa, ON, Canada) for cooking and freeze-drying. We used identical pre- and post-drying parameters, with all analytical determinations performed at the University of Ottawa, such that the two processing lines differed only in the drying step (Figure 1). The ingredients used to produce the cupcakes: commercial gluten-free all-purpose flour blend (Ardent Mills, Mississauga, ON, Canada), milk, eggs, baking powder, sugar, salt, and vegetable oil, were purchased from a local supermarket (Ottawa, ON, Canada).

### 2.2. Preparation of Cranberry Beans Flour

**Figure 2:**
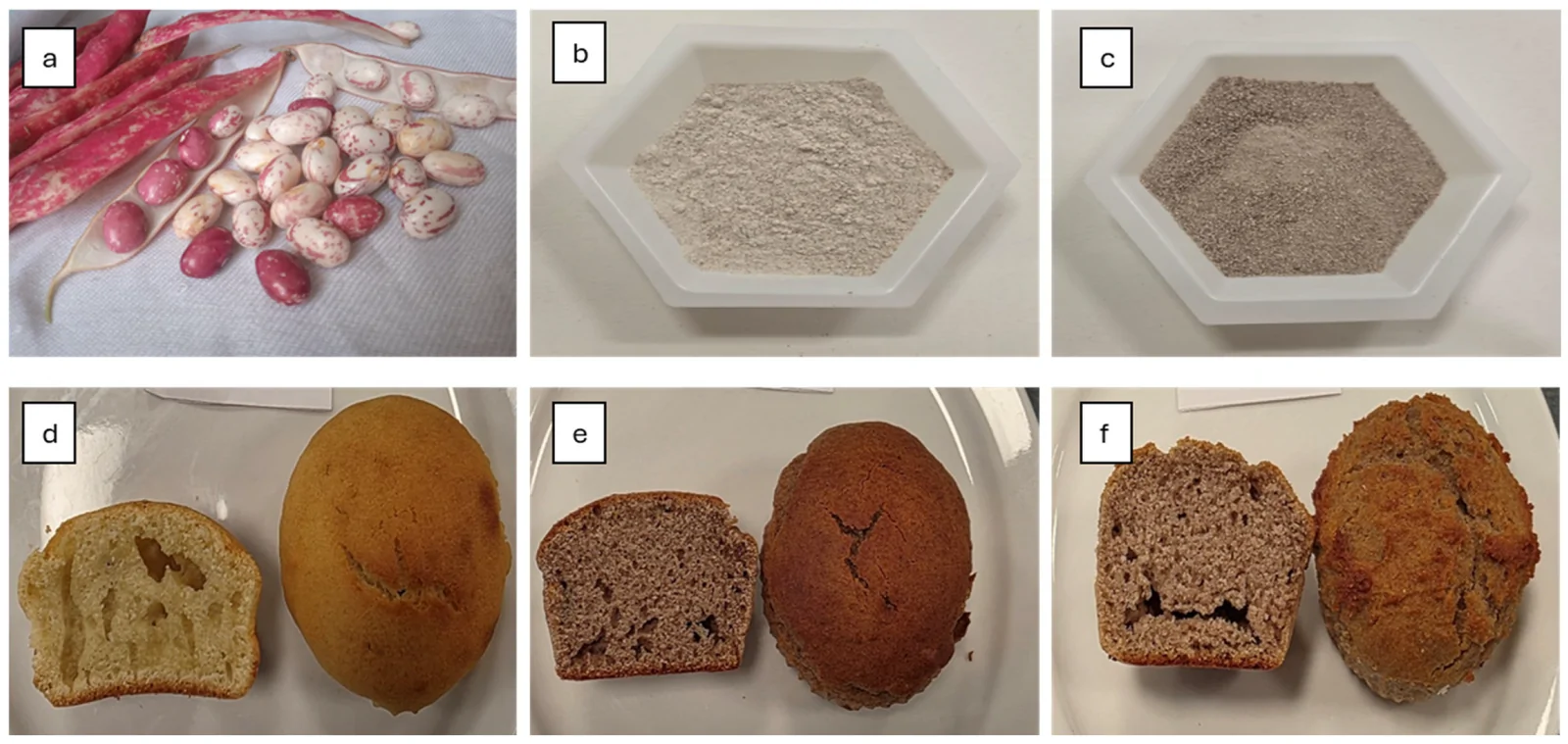
Visual characterization of raw bean, bean flours, and gluten-free cupcakes. (**a**) raw cranberry beans; (**b**) oven-dried cranberry bean flour; (**c**) freeze-dried cranberry bean flour. Cross-sections of gluten-free cupcakes: (**d**) control cupcakes formulated with commercial gluten-free all-purpose flour; (**e**) cupcakes formulated with oven-dried cranberry bean flour; (**f**) cupcakes formulated with freeze-dried cranberry bean flour.

As shown in Figure 1, CBF was produced using two drying methods: OD and FD. The raw cranberry beans (Figure 2a) were sorted and rinsed then soaked in water overnight at room temperature. Following soaking, the beans were drained and cooked in fresh water at 100 °C for 25 min using a 1:2 (*w*/*v*) bean-to-water ratio. The cooking was conducted without the addition of salt or any other additives to ensure the purity of the samples. Cooked beans were blended with the cooking water at a ratio of 1.4:1 (*w*/*v*) to obtain a uniform paste. For the first batch, drying was performed in a convection oven at 40 °C for 12 h until the moisture content was less than 10%. The second batch was pre-frozen and subjected to vacuum freeze-drying using a BUCHI Lyovapor L-200 (BUCHI Labortechnik AG, Flawil, Switzerland) at −50 °C and a pressure of 1 mbar until complete dryness was achieved. The dried samples were ground using a blender (Magic Bullet, 250 W, NutriBullet LLC, Los Angeles, CA, USA) for 30 s and sieved through a fine-mesh stainless steel strainer (Thinkkitchen, Stokes Inc., Mont-Royal, QC, Canada) to obtain flours (Figure 2b,c). The bean flours were vacuum sealed in pouches using a vacuum packaging machine (Santa Ana, Atmovac, Eurodib Inc., Boucherville, QC, Canada) and stored in sealed polyethylene bags at room temperature until use.

### 2.3. Flour Functional Properties

#### 2.3.1. Water Absorption Capacity (WAC) and Oil Absorption Capacity (OAC)

The Water Absorption Capacity (WAC) was determined according to the method described by Anderson et al. [30] with slight modifications. Briefly, 1.0 g of flour was dispersed in 10 mL of distilled water, vortexed vigorously to ensure a uniform suspension, and incubated in a water bath at 37 °C for 30 min before centrifugation at 3000× *g* for 10 min. The supernatant was carefully discarded, and the wet sediment was weighed. The Oil Absorption Capacity (OAC) was determined following the method of Lawal [31] with minor modifications. Briefly, 1.0 g of flour was combined with 6 mL of commercial canola oil (Saporito Foods, Markham, ON, Canada) in a 15 mL centrifuge tube and homogenized using a vortex for 30 s. The mixture was left to stand at room temperature for 30 min and subsequently centrifuged at 3000× *g* for 30 min. Following centrifugation, the unbound oil was carefully decanted, and the mass of the remaining oil-saturated sediment was recorded. Both WAC and OAC were calculated using the following equation:

(1) $$\mathrm{WAC}\;\mathrm{or}\;\mathrm{OAC}\;(g/g)\;=\;\frac{W_{1}\;-\;W_{2}}{W_{2}}$$

where *W*_1_ is the weight of the wet pellet (g) and *W*_2_ is the weight of the dry sample (g).

The results were expressed as grams of water or oil retained per gram of dry sample. All measurements were performed in triplicate for each flour preparations (*n* = 3).

#### 2.3.2. Particle Size Analysis

Particle size distribution was determined by laser diffraction (Mastersizer 3000, Malvern Panalytical, Malvern, UK) using the wet dispersion method with deionized water as the dispersant. The optical properties were set with a refractive index (RI) of 1.53 and an absorption index of 0.1. Measurements were performed in triplicate.

#### 2.3.3. Moisture Content

Moisture content was determined using an infrared moisture analyzer (MA37, Sartorius Lab Instruments GmbH & Co. KG, Göttingen, Germany). Samples were heated at 130 °C in standard drying mode until a constant weight was achieved, with the moisture percentage recorded automatically by the device. Measurements were performed in triplicate (*n* = 3).

### 2.4. Flour Proximate Composition

Protein content (N × 6.25) was determined by the Kjeldahl method (AOAC 990.03). Fat content was determined via Soxhlet extraction (AOAC 920.39). Ash content was measured by incineration in a muffle furnace (AOAC 942.05). Total dietary fiber was determined using the enzymatic-gravimetric method (AOAC 2022.01). Total starch content was quantified using the AOAC 996.11 method. Total carbohydrate content was calculated by difference: 100 − (moisture + protein + fat + ash). Caloric content was calculated using Atwater factors (4 kcal/g protein, 9 kcal/g fat, 4 kcal/g carbohydrate). All results were expressed on a dry weight basis to ensure comparability between the oven-dried (OD) and freeze-dried (FD) samples. Mineral content (sodium, calcium, magnesium, potassium, phosphorus, iron, and zinc) was determined by Inductively Coupled Plasma-Atomic Emission Spectroscopy (ICP-AES) (EPA Method 6010), performed at A&L Canada Laboratories Inc. (London, ON, Canada) according to standard plant analytical protocols. All results were expressed on a dry matter basis. Proximate composition and mineral analyses were performed in duplicate (*n* = 2) for each flour type.

### 2.5. Determination of Total Phenolic Content

Total phenolic content (TPC) was determined using the Folin–Ciocalteu colorimetric method with gallic acid as the standard [25]. The results were calculated from the standard curve and expressed as gallic acid equivalents (GAE) per gram of dry sample. Each flour type was analyzed in triplicate (*n* = 3).

### 2.6. Cupcake Formulation and Preparation

Three different gluten-free cupcakes were prepared. The first formulation, utilized as the control, was prepared with 100% commercial gluten-free flour blend (Gluten Free 1-To-1 All Purpose Flour Blend, Ardent Mills, Mississauga, ON, Canada) consisting of white rice flour, potato starch, tapioca starch, whole sorghum flour, brown rice flour, whole quinoa flour, and xanthan gum in descending order of weight. The second formulation was prepared using 100% OD bean flour, while the third formulation was made with 100% FD bean flour. The bean-based cupcakes were prepared using the same recipe; however, the control formulation made with commercial gluten-free flour contained a lower amount of liquid ingredients to ensure a batter consistency appropriate for cupcakes as presented in Table 1. The batter was portioned into molds and baked in a preheated oven (Bertazzoni S.p.A., Guastalla, Italy) at 180 °C for 20 min. Following baking, the cupcakes were cooled to room temperature before undergoing physical and sensory analysis (Figure 2d–f).

### 2.7. Color Analysis

Cupcake crumb color was evaluated after 24 h of baking using a HunterLab ColorFlex EZ spectrophotometer (Hunter Associates Laboratory Inc., Reston, VA, USA) calibrated using standard black and white tiles before measurement. Color parameters were recorded in the CIE LAB coordinates: L*: Lightness (ranging from 0 = black, 100 = white), a*: Redness (+)/Greenness (−), and b*: Yellowness (+)/Blueness (−). The Browning Index (BI) was calculated using the equation: BI = 100 − L* according to Guadalupe-Moyano et al. [26]. Triplicate measurements were performed for each sample.

### 2.8. Moisture Content, Volume, and Texture Profile Analysis (TPA) of Cupcakes

Cupcake moisture content was determined as described in Section 2.3.3. The weight (g) and height (cm) of the samples were recorded. The volume was determined as the ratio of the cupcake height to its weight as described by Ghoname and El-Gwad [32]. The textural properties of the cupcakes were evaluated using a Brookfield Ametek CTX Texture Analyzer (Brookfield, Middleboro, MA, USA) equipped with TA-25/1000 cylindrical probe [2]. Cupcake samples were subjected to a double compression cycle at a test speed of 1 mm/s to reach a 40% compression of their original height (trigger load 5.0 g). Parameters including hardness, resilience, cohesiveness, springiness, and chewiness were calculated. All measurements were conducted in triplicate.

### 2.9. Storage Behavior

Beans cupcakes were stored in sealed polyethylene bags at room temperature (22 ± 1 °C) for 7 days to monitor storage behavior. Moisture content and TPA parameters were measured at Days 1 and 7 as described in Section 2.8.

### 2.10. Sensory Evaluation

Sensory evaluation was conducted in the Sensory Analysis Room at the Faculty of Health Sciences, University of Ottawa (Ottawa, ON, Canada). A panel of 45 untrained participants (32 females, 13 males; age range 20–63 years) was recruited from the University of Ottawa community by posters. Informed consent was obtained from all panelists, and they were screened for allergies or intolerances to any of the ingredients used in the cupcakes (eggs, beans, and milk). Volunteers reporting such an allergy were excluded. Cupcakes were baked on the day of the evaluation and cooled to room temperature before serving. Cupcake samples were presented in individual sensory booths under controlled conditions (22 °C, white fluorescent lighting, odor-free environment). Room-temperature water was provided as a palate cleanser, and panelists were instructed to use it between samples. Each sample of the three formulations (control, OD and FD; approximately 10 g each) was coded with three-digit random numbers and presented in a randomized order. Panelists evaluated the samples for specific attributes including appearance, aroma, texture, and flavor along with overall acceptability using a 9-point hedonic scale. On this scale, 1 represented “dislike extremely,” to 9 represented “like extremely.” A 5-point Just-About-Right (JAR) scale was used to evaluate bean flavor intensity and aftertaste, ranging from 1 (“much too weak”) to 5 (“much too strong”), with 3 representing the ideal level. For presentation, JAR responses were grouped into three categories: “too weak” (1–2), “just-about-right” (3), and “too strong” (4–5). Results were expressed as percentages to evaluate the impact of cranberry bean flour on the sensory profile of the cupcakes.

### 2.11. Statistical Analysis

Data are expressed as mean ± standard deviation (SD). Statistical analyses were performed using GraphPad Prism v8.4.3. Statistical significance was determined using Student’s *t*-test, one-way analysis of variance (ANOVA) or two-way ANOVA, as appropriate followed by Tukey’s post-hoc test. Differences were considered statistically significant at *p* < 0.05.

## 3. Results and Discussion

### 3.1. Effect of Oven-Drying or Freeze-Drying on the Physicochemical Properties of Cranberry Bean Flours

**Figure 3:**
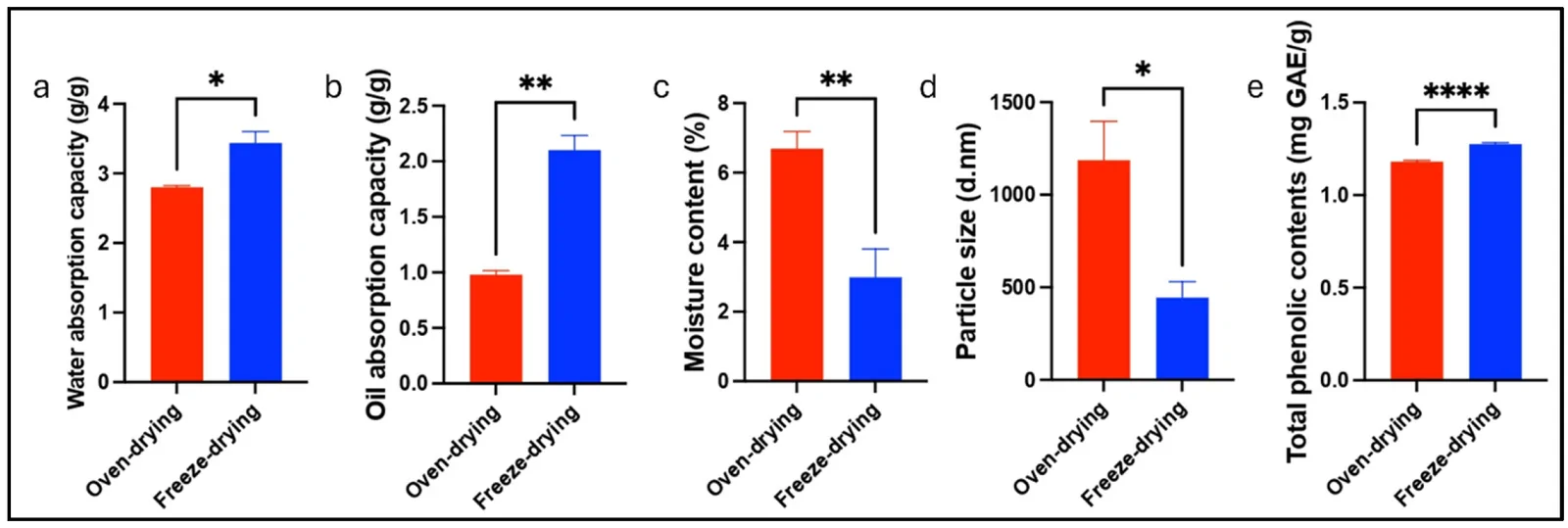
Physicochemical properties and total phenolic compound content of cranberry bean flour prepared by oven-drying and freeze-drying. (**a**) water absorption capacity (g/g dry weight), (**b**) oil absorption capacity (g/g dry weight), (**c**) moisture content (%), (**d**) particle size (nm), and (**e**) total phenolic compounds (mg GAE/g dry weight). GAE, gallic acid equivalents. Values are expressed as mean ± standard deviation (*n* = 3). Statistical significance was considered at *p* < 0.05. * *p* < 0.05; ** *p* < 0.01; **** *p* < 0.0001.

The physicochemical properties and total phenolic content of CBF as influenced by the drying method are presented in Figure 3.

#### 3.1.1. Water and Oil Absorption Capacities

Water absorption capacity (WAC) is a crucial functional parameter, as it influences the texture, moisture content, and starch retrogradation of food products [33]. CBF exhibited high WAC values (2.80 ± 0.02 g/g for OD flour and 3.44 ± 0.16 g/g for FD flour) relative to wheat flour (0.5–0.7 g/g) and rice flour (0.5 g/g) reported in the literature [16,34], consistent with previous findings showing that higher concentrations of bean flour are associated with greater water absorption [33]. The elevated WAC of bean flour is likely attributable to its high protein and arabinoxylan content, which promote water binding and retention [33,34]. FD resulted in significantly higher absorption indices than OD: WAC increased 1.2-fold (from 2.80 ± 0.02 g/g to 3.44 ± 0.16 g/g; *p* = 0.02), an effect that can be attributed to the lyophilization process, during which water is removed through sublimation, generating a porous structure that facilitates water entry and enhances hydration properties [26].

Oil absorption capacity (OAC) reflects the ability of proteins to bind and retain fat within food formulations, and serves as an important indicator of product stability during storage, particularly with respect to oxidative rancidity [33]. Bean flour OAC in the present study was higher than values reported for wheat (0.6 g/g) and rice flours (0.5 g/g) [16]. OAC exhibited a 2.1-fold increase in FD samples (2.10 ± 0.13 g/g) relative to OD samples (0.98 ± 0.03 g/g; *p* = 0.003), consistent with findings by Que et al. [25] for pumpkin flour, where FD (2.38 ± 0.04 mL/g) produced significantly higher OAC than hot-air drying (1.08 ± 0.02 mL/g). This enhancement is likely driven by the combined effects of reduced particle size, and the higher starch and protein contents associated with the FD process [33,35].

#### 3.1.2. Moisture Content and Particle Size

Flour moisture content is a major determinant of shelf life [32]. FD yielded a significantly lower moisture content (2.99 ± 0.8 vs. 6.69 ± 0.5%; *p* = 0.005) than OD, which may contribute to improved shelf stability by preventing microbial spoilage [32]. These findings are consistent with a recent study reporting moisture values of 10.34 ± 1.15 and 6.36 ± 0.42 for OD and FD cranberry bean flours, respectively [28]. FD also produced a smaller particle size (445 ± 86 vs. 1187 ± 210 nm; *p* = 0.02), which is relevant given that particle size directly influences the digestibility and overall baked product quality of legume flours [4].

#### 3.1.3. Total Phenolic Content

CBF contains relatively high levels of total phenolic compounds (TPC), including catechins and proanthocyanidins, known for their antioxidant properties and multiple health benefits [18]. TPCs also contribute to gut health by strengthening the intestinal mucosal barrier and attenuating oxidative stress [36]. Raw cranberry beans have been reported to contain a TPC of 3 mg GAE/g [17]. In the present study, CBF exhibited TPC values of 1.18 ± 0.008 mg GAE/g (OD) and 1.23 ± 0.006 mg GAE/g (FD), both of which were notably higher than values reported for other functional flours, including roasted apricot kernel flour (0.00458 mg/g), barley flour (0.88 mg/g), and various sweet potato flours: raw (0.0479–0.0629 mg/g), steamed (0.1013–0.3257 mg/g), and kneaded (0.0836–0.2904 mg/g) [25]. Öncel [5] demonstrated that incorporating only 30% CBF into bread formulations increased TPC from 0.41 to 2.49 mg GAE/100 g. In the present study, TPC was significantly higher in FD flour (1.23 ± 0.006, *p* = 7.5 × 10^−5^) than in the OD flour, in contrast to the findings of Que et al. [25] who reported higher TPC in hot-air-dried pumpkin flour than in FD pumpkin flour. This discrepancy likely reflects the high sensitivity of phenolic compounds to specific drying methods, temperature conditions, and flour type employed. Taking together, these results confirm that the drying technique significantly influences the physicochemical characteristics of CBF, with FD yielding superior overall functional properties compared with OD.

### 3.2. Effect of Oven-Drying or Freeze-Drying on the Nutritional Properties of Cranberry Bean Flours

The proximate composition of the dry CBF (per 100 g) prepared by OD and FD is presented in Table 2. FD yielded a higher caloric value (401 ± 1.6 Cal) than OD (392 ± 0.9 Cal; *p* = 0.02). Although the difference was not statistically significant, FD yielded higher levels of protein (24.4 ± 2 vs. 23.4 ± 1.7 g) and fat (2 ± 0.5 vs. 1.7 ± 0.03 g). These findings are consistent with a recent study on CBF in which FD produced significantly higher lipid (2.53 ± 0.16 g) and protein (23.99 ± 0.41 g) contents compared with OD (1.90 ± 0.50 g and 21.73 ± 0.87 g, respectively) [28]. OD and FD had comparable contents of carbohydrate (70.7 ± 1.4 vs. 71.2 ± 2.7 g), starch (30.7 ± 3.3 vs. 39.4 ± 0.7 g), fiber (22.5 ± 1.6 vs. 21.5 ± 0.1 g), and sugars (6.6 ± 0.5 vs. 6.9 ± 0.1 g). Overall, the OD method retained higher concentrations of essential minerals, with potassium (1701 ± 1.4 vs. 945 ± 91.9 mg), iron (6.4 ± 0.4 vs. 4.8 ± 0.3 mg), phosphorus (501 ± 1.4 vs. 300 ± 28.3 mg), and zinc (3 ± 0.3 vs. 1.7 ± 0.3 mg) being significantly higher than in FD.

Despite these differences, CBF represents a nutrient-dense, gluten-free ingredient with considerable potential to enhance the nutritional profile of food products. As reported by Öncel [5], enriching gluten-free bread with 30% CBF increased protein content from 2.75% to 10.14% and total dietary fiber from 1.28% to 2.63%, with remarkable mineral gains including increases in magnesium, calcium, and iron of approximately 2707%, 1421%, and 383%, respectively. A comparable dietary fiber abundance (21–24 g/100 g) has been observed in both Chinese and Brazilian bean varieties [37]. In Canada, food products containing at least 6 g of dietary fiber per serving are classified as “very high in fiber” [38]. The CBF characterized in the present study contained approximately 21–23 g of dietary fiber per 100 g, representing nearly four times the threshold for this classification, thus underscoring its richness in this nutrient. Dietary fiber promotes greater microbiota diversity, inhibits the growth of pathogenic microorganisms, and may influence mental health through the microbiota–gut–brain axis [39]. Furthermore, the fermentation of dietary fiber enhances immune function through microbiota-derived metabolites such as short-chain fatty acids (SCFAs) [39]. Collectively, these findings position cranberry beans as a rich source of macro- and micronutrients essential for health, and as an important candidate for replacing existing gluten-free flours, which are generally of low nutritional value.

### 3.3. Effect of Oven-Drying or Freeze-Drying on the Quality Characteristics of Gluten-Free Cranberry Bean Cupcakes

Having characterized the physicochemical and nutritional properties of CBF, we next investigated how these differences influenced the quality characteristics of gluten-free cupcakes.

#### 3.3.1. Volume and Crumb Color

The volume of the gluten-free cupcakes after 24 h of baking is presented in Table 3. A significant difference was observed in the volume (height/weight ratio) across formulations (*p* = 0.01), with control cupcakes exhibiting the highest volume (0.09 ± 0.008). Optimization of the formulation and the inclusion of additional structural ingredients could further improve the volume for cranberry bean cupcakes. Cupcake crumb color was assessed using the L*, a*, and b* parameters. As shown in Table 3, control cupcakes were characterized by significantly higher lightness (L* = 68.25 ± 3.02), yellowness (b* = 29.0 ± 0.88), and lower redness (a* = 4.98 ± 0.49) compared with bean-flour cupcakes, which is consistent with the predominantly white rice flour composition of the control and the well-documented tendency of conventional gluten-free products to exhibit pale coloration due to reduced Maillard reactions [3]. These findings are in agreement with previous research confirming that control samples formulated with white rice flour exhibit higher L* and b* values than red bean-based samples [32]. According to Shevkani and Singh [15], the addition of kidney bean flour to muffins decreased crumb L* values and increased a* values, effects attributed to the inherent color characteristics of the flour. Redness (a*) was significantly higher in both bean-flour formulations than in the control (*p* = 0.0006), likely because cranberry bean flour contains phytopigments such as anthocyanins and flavonoids that may form chromophore structures upon heating [5]. The relationship between colored bean flour concentration and product darkness is well established in the literature [4,5,33]. The OD cupcakes exhibited the highest browning index (51.97 ± 0.68; *p* = <0.0001). Given that all cupcake batters were subjected to identical cooking conditions, the darker color of OD and FD cupcakes is attributed to their darker flour, likely reflecting greater Maillard browning driven by the higher protein content of these flours [26,33]. Between bean treatments, FD cupcakes were significantly lighter (L* = 53.42 ± 0.9) than OD cupcakes (L* = 48.03 ± 0.7; *p* = 0.018), consistent with results reported by Guadalupe-Moyano et al. [26], who observed that FD banana flour produced significantly lighter bread than its OD counterpart. Color is a crucial quality attribute for cupcakes, as it strongly influences consumer acceptability [32,33]. Notably, Contini et al. [4] incorporated cocoa powder into bean-based muffins to improve their acceptability.

#### 3.3.2. Moisture Content and Texture Profile Analysis

**Figure 4:**
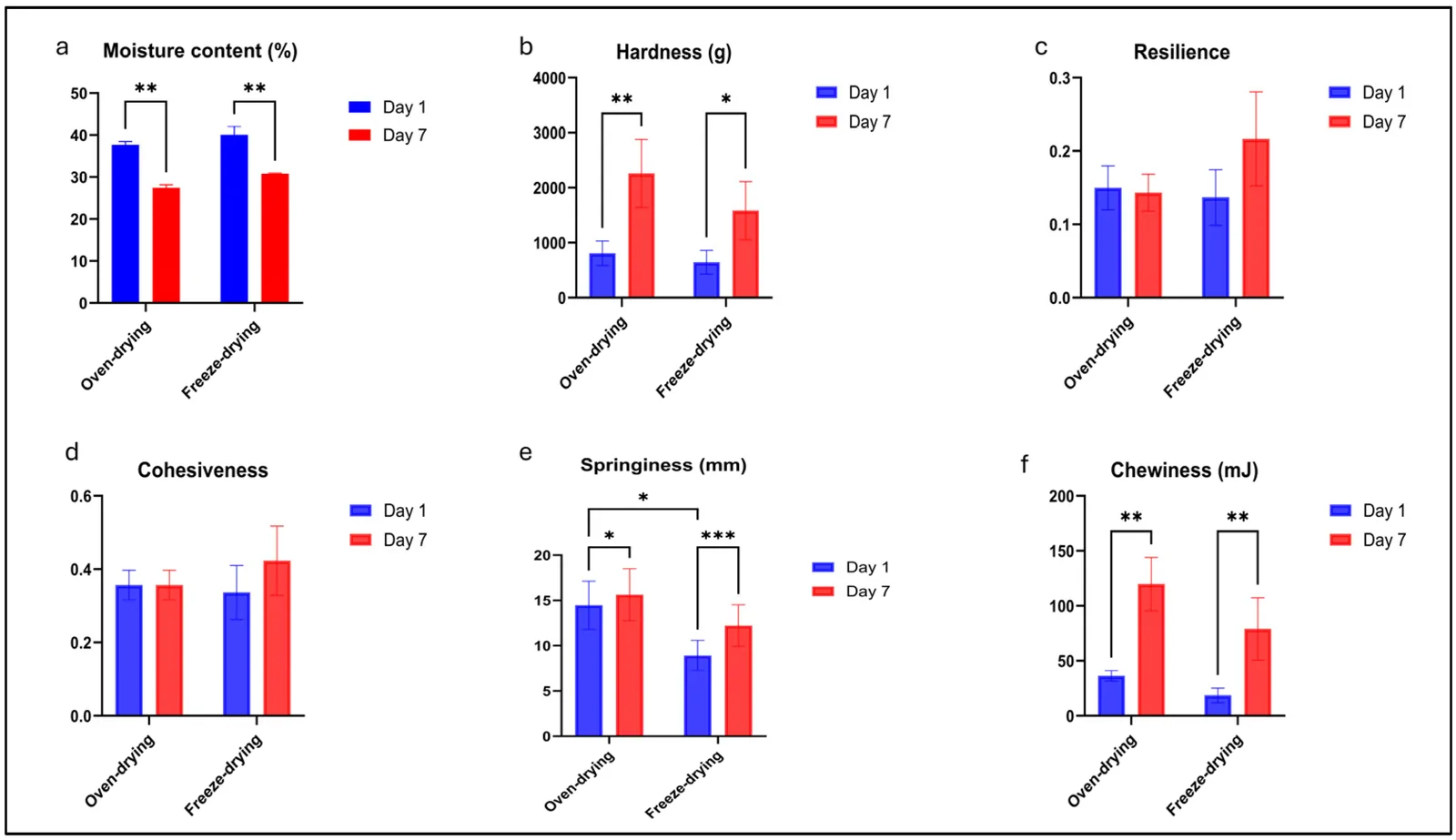
Physicochemical properties of gluten-free cranberry bean cupcakes. (**a**) moisture content (%) after 1 and 7 days of storage. Textural properties after 1 and 7 days of storage including (**b**) hardness, (**c**) resilience, (**d**) cohesiveness, (**e**) springiness (mm), and (**f**) chewiness (mJ). Values are expressed as mean ± standard deviation (*n* = 3). Statistical significance was considered at *p* < 0.05. * *p* < 0.05; ** *p* < 0.01; *** *p* < 0.001.

Moisture content and texture profile analysis (TPA) parameters were evaluated on Day 1 post-baking and after 7 days of storage (Day 7) to assess the storage-related changes over time. As the OD and FD cupcakes had identical formulations, these parameters were compared only between the bean formulations. The control formulation was excluded from these analyses because it required a lower liquid content to achieve a comparable batter consistency. Results are presented in Figure 4. On day 1, moisture content did not differ significantly between the two bean-flour formulations (37.68 ± 1.4% for OD; 40.06 ± 3.4% for FD), indicating that the drying method applied to the flour did not alter the amount of water retained in the baked crumb. Both formulations reached moisture levels higher than those commonly reported for control gluten-free cupcakes, attributable to the high fiber and protein contents in bean flour, which enhance water retention [4,16,33].

Over the 7-day storage period, moisture content declined significantly in both formulations, yet OD and FD cupcakes still retained high moisture levels at Day 7 (1.37-fold reduction to 27.42 ± 1.3%, and 1.31-fold reduction to 30.63 ± 0.53%, respectively) with no significant difference between treatments. These findings are consistent with a previous study reporting a progressive decrease in moisture content during storage of bean flour-based baked products [33].

TPA revealed no significant differences in hardness, resilience, cohesiveness, or chewiness between the two bean flour formulations, indicating comparable crumb firmness and internal structure (Figure 4). The low hardness values observed in bean-based cupcakes may be attributed to the higher protein and fiber contents of CBF, that enhance water retention and contribute to a less compact crumb structure. This is consistent with findings by Öncel [5], who reported lower hardness in bread containing 30% cranberry bean flour relative to the control formulation. Resilience, reflecting the crumb’s ability to recover its original shape after deformation, did not differ significantly between OD (0.15 ± 0.03) and FD (0.14 ± 0.04) cupcakes, indicating similar elastic recovery [16]. Cohesiveness, describing the internal structural strength of the crumb and its ability to withstand a second deformation without fracturing [16], was also comparable between OD (0.36 ± 0.04) and FD (0.34 ± 0.07) cupcakes. This is consistent with the high moisture content of bean-based cupcakes, which yields a less compact crumb. Previous findings showed that control samples formulated with 100% rice flour exhibited higher cohesiveness than cupcakes containing 20% bean flour [16].

Both bean formulations exhibited high springiness values, which is indicative of better-quality cupcake with an aerated and elastic crumb structure [16]. Kidney bean pod powder-enriched gluten-free cakes have similarly shown increased springiness alongside lower hardness and chewiness [14]. Springiness was the only textural parameter significantly affected by the drying method: OD cupcakes showed higher values (14.47 ± 2.65 mm) than FD cupcakes (8.91 ± 1.68 mm; *p* = 0.023), indicating greater elasticity in the oven-dried formulation. Chewiness, defined as the energy required to masticate the sample to a swallowable state [16], was not significantly different between treatments but was numerically lower in FD (15.59 ± 6.69 mJ) than in OD (39.06 ± 7.9 mJ) cupcakes, indicating a lower energy requirement. While the drying method had a statistically significant effect only on springiness, the other TPA parameters also showed numerical variation between treatments. These observations are broadly consistent with findings by Guadalupe-Moyano et al. [26], who reported significant differences in resilience, cohesiveness, and chewiness, but not hardness and springiness, between OD and FD flour-based breads.

#### 3.3.3. Storage Behavior

Between Day 1 and Day 7, storage behavior analysis revealed significant increase in hardness, springiness, and chewiness in both formulations, and no significant difference between OD and FD cupcakes was detected for any textural parameter at Day 7 (Figure 4). The drying method did not significantly affect textural changes during storage, indicating similar storage behavior for OD and FD bean flour. Importantly, both formulations retained more than 27% moisture and remained soft and elastic after 7 days. This behavior is consistent with previous reports on bean flour-based baked products: Cakir et al. [16] found that kidney bean muffins showed the lowest chewiness and resilience values on the seventh day of storage, and Sanfilippo et al. [33] reported that a 10% bean flour sample exhibited lower hardness than the control after 6 days of storage. Proteins may contribute to reduced staling by interacting with starch and limiting retrogradation within food matrices [40].

Rapid staling is a major challenge in gluten-free cupcakes, which tend to lose moisture, softness, and crumb elasticity shortly after baking due to the absence of gluten’s natural structural network [2,16]. Although direct verification of this anti-staling effect would require comparison with a matched gluten-free control formulation, these findings of the present study suggest that bean-based formulations, for both drying methods, offer a promising solution, as they not only improve the nutritional quality of gluten-free cupcakes but also help maintain desirable physicochemical properties, including softness and elasticity, during storage.

### 3.4. Effect of Oven-Drying or Freeze-Drying on the Sensory Properties of Gluten-Free Cranberry Bean Cupcakes

**Figure 5:**
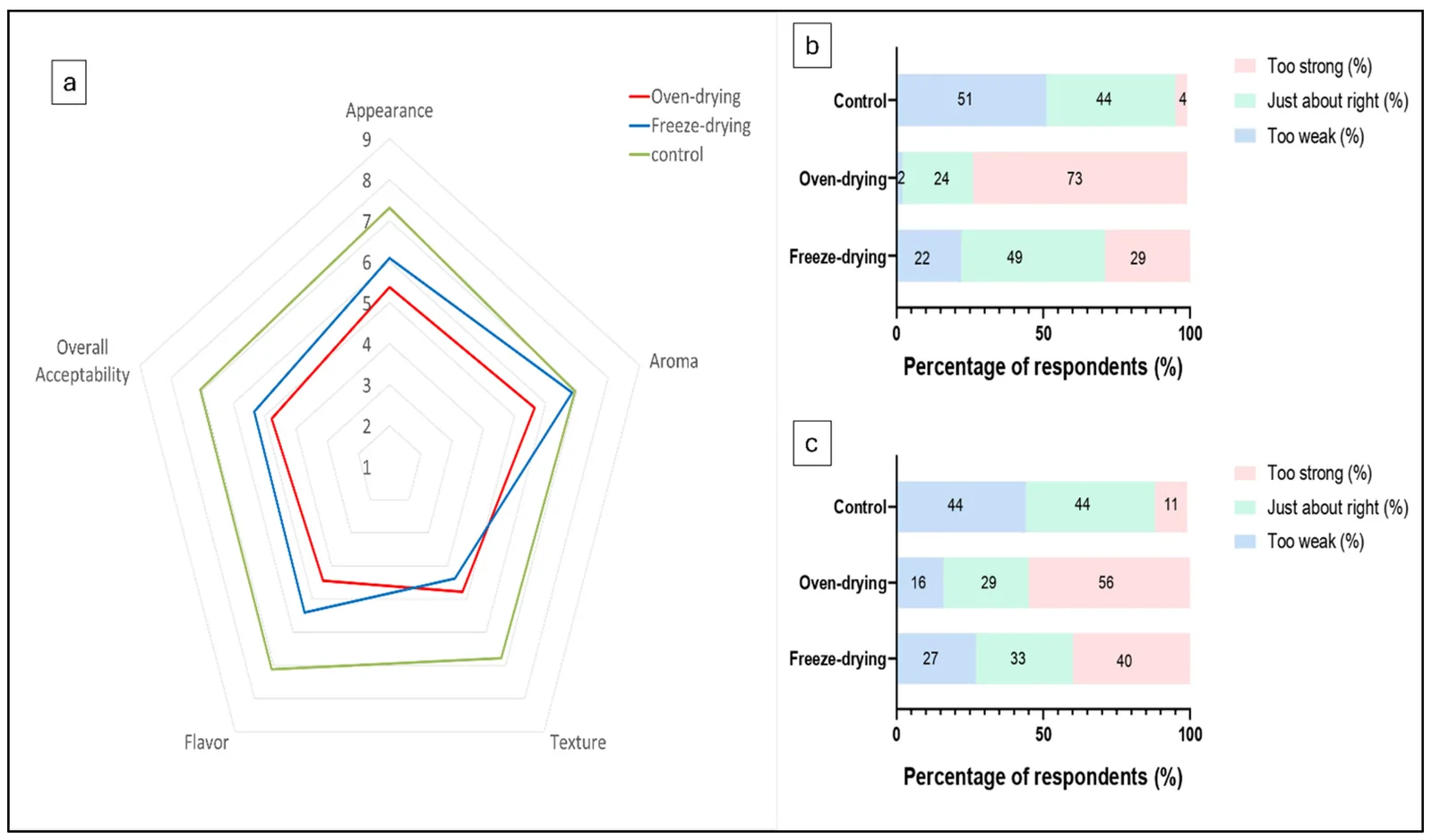
Sensory profile of gluten-free cupcakes. (**a**) Radar chart representing mean consumer liking scores on a 9-point hedonic scale; (**b**) Just-about-right (JAR) frequency distribution for bean flavor intensity; and (**c**) JAR frequency distribution for aftertaste (*n* = 45).

A sensory evaluation was conducted with 45 panelists aged 20–63 years (with 71% female) to assess consumer acceptability of OD and FD cranberry bean cupcakes relative to the control formulation. Sensory properties were evaluated using a 9-point hedonic scale across five attributes (appearance, aroma, texture, flavor, and overall acceptability) and summarized in a radar chart (Figure 5a). Higher sensory scores were recorded for the control sample, reflecting the sensory familiarity and optimized formulation characteristics of the commercial gluten-free product relative to the bean-based samples. This is consistent with the literature, where higher proportions of rice flour have been associated with higher sensory scores [6,12], and with well-established influence of appearance and color on product acceptability prior to taste evaluation [32]. Cupcakes prepared with OD flour received sensory scores ranging from “dislike slightly” to “like slightly,” whereas cupcakes made with FD flour were rated from “dislike slightly” to “like moderately”, in line with sensory scores reported by Chompoorat et al. [12] for red kidney bean cupcakes.

Bean-based cupcakes did not differ significantly in appearance, texture, or overall acceptability; however, aroma differed significantly between treatments, with FD flour receiving a higher mean score (“Like Moderately”, 6.84 ± 1.1) than OD flour (“Like Slightly”, 5.64 ± 1.7; *p* = 0.0002). A similar trend was observed for flavor, which improved from “Dislike Slightly” (4.44 ± 2.2) for OD flour to a neutral response (“Neither”, 5.40 ± 1.6) for FD flour (*p* = 0.03). A previous study evaluating crackers formulated with navy bean powder reported low consumer acceptability due to the pronounced “beany flavor” perceived by panelists, with a flavor score of 3.9 on a 9-point hedonic scale (*n* = 20) [41]. In contrast, FD bean flour cupcakes in the present study achieved a higher mean flavor score of 5.4 ± 1.6 on the same scale (*n* = 45), suggesting that FD bean flour may be successfully incorporated into acceptable baked products. According to Chompoorat et al. [12], vacuum treatment and soaking are effective strategies for reducing the characteristic beany flavor of bean flour. Notably, the use of FD flour resulted in no significant difference in the aroma relative to the control (6.84 ± 1.1 vs. 6.93 ± 1.2; *p* = 0.9), indicating that FD effectively masked or neutralized the beany aroma.

Bean flavor intensity and aftertaste were further assessed using a five-point Just-About-Right (JAR) scale, enabling panelists to indicate whether each attribute was perceived at an appropriate intensity or was too weak or too strong. The FD formulation had a significant effect on bean flavor intensity compared with the OD. For FD cupcakes, 49% of panelists rated the bean flavor intensity as “just-about-right,” while only 29% considered it “too strong.” In contrast, 73% of panelists rated the bean flavor intensity of OD cupcakes as “too strong” (*p* = 0.017) (Figure 5b), indicating that FD largely balanced the residual bean flavor at an acceptable level. No significant difference was observed for aftertaste between treatments; however, the OD formulation recorded the highest “too strong” rate (56%) across all samples for this attribute (Figure 5c), consistent with the bean flavor intensity findings and suggesting that OD cranberry bean flour may generate stronger flavor compounds during processing. Despite these observations, it is worth noting that Lutomia et al. [42] reported that the perceived bean flavor in precooked bean snacks increased the likelihood of future purchase, reflecting consumer interest in bean-based food products driven by their recognized nutritional value and potential health benefits [6].

### 3.5. Limitations

Oven- and freeze-drying were performed at different facilities, as both techniques were not available at the same site. Although the source material, pre- and post-drying processing steps, and all analytical determinations were standardized to a single operator and a single analytical site, facility-level factors associated with the two drying locations cannot be fully excluded. Moisture and TPA comparisons were restricted to the OD and FD formulations, which were prepared identically. The control required less liquid to achieve a suitable batter consistency, given the lower water absorption capacity of the commercial gluten-free blend. Storage behavior was assessed only over a 7-day period and only through moisture content and texture profile analysis, so no anti-staling effect can be claimed from the present data. Sensory evaluation was based on a limited sample size (*n* = 45) from a non-representative panel, and these results should not be generalized to broader consumer populations. These aspects are proposed as priorities for future research.

## 4. Conclusions

This study demonstrated that the drying method significantly influences the physicochemical and nutritional properties of CBF, as well as the quality and sensory characteristics of gluten-free cranberry bean cupcakes. FD significantly increased water and oil absorption capacities, reduced particle size, and enhanced TPC compared with OD. While both drying methods yielded comparable dietary fiber, fat, carbohydrate, starch, sugars, and protein, FD had higher caloric values. Despite these differences, both CBF preparations demonstrated strong potential as nutrient-rich ingredients capable of improving the macro- and micro-nutrient profile of gluten-free baked products.

In cupcakes, FD produced significantly lighter products with lower browning intensity. Bean-based cupcakes exhibited high moisture content. Over 7 days of storage, the two bean formulations showed comparable storage behavior, retaining high moisture and a soft, elastic crumb. Sensory analysis further showed that FD cupcakes had significantly lower bean flavor intensity and higher aroma liking than OD formulations, suggesting improved consumer acceptability.

Overall, freeze-dried CBF shows strong potential as a functional ingredient for producing nutritionally improved gluten-free cupcakes with favorable texture and desirable sensory properties. Nevertheless, further optimization of formulation and processing conditions is warranted to improve the nutritional, physicochemical, and sensory properties of the final product. Future studies should also investigate the health benefits associated with the regular consumption of these bean-based cupcakes, including their effects on gut microbiota composition and metabolic health markers.

## Figures and Tables

**Table 1:** Ingredients and amounts used in cupcakes formulation.

Ingredients	Oven-Dried Bean Flour Cupcake	Freeze-Dried Bean Flour Cupcake	Control Cupcake
Commercial flour (g)	-	-	100
Oven-dried bean flour (g)	100	-	-
Freeze-dried bean flour (g)	-	100	-
Eggs (unit)	2	2	2
Sugar (g)	60	60	60
Milk (mL)	125	125	80
Oil (mL)	30	30	25
Baking powder (g)	3	3	3
Salt (g)	0.6	0.6	0.6

**Table 2:** The proximate composition (dry basis, per 100 g) of precooked cranberry bean flour prepared by oven-drying and freeze-drying.

Drying Methods	Oven-Drying (Mean ± SD)	Freeze-Drying (Mean ± SD)
Calories (Cal)	392 ± 0.9 ^a^	401 ± 1.6 ^b^
Fat (g)	1.7 ± 0.03 ^a^	2.0 ± 0.5 ^a^
Carbohydrates (g)	70.7 ± 1.4 ^a^	71.2 ± 2.7 ^a^
Starch (g)	30.7 ± 3.3 ^a^	39.4 ± 0.7 ^a^
Fiber (g)	22.5 ± 1.6 ^a^	21.5 ± 0.1 ^a^
Sugars (g)	6.6 ± 0.5 ^a^	6.9 ± 0.1 ^a^
Protein (g)	23.4 ± 1.7 ^a^	24.4 ± 2 ^a^
Sodium (Na) (mg)	21 ± 1.3 ^a^	21 ± 1.3 ^a^
Potassium (K) (mg)	1701 ± 1.4 ^a^	945 ± 91.9 ^b^
Calcium (Ca) (mg)	115 ± 21.2 ^a^	110 ± 14.1 ^a^
Iron (Fe) (mg)	6.4 ± 0.4 ^a^	4.8 ± 0.3 ^b^
Magnesium (Mg) (mg)	155 ± 21.2 ^a^	105 ± 21.2 ^a^
Phosphorus (P) (mg)	501 ± 1.4 ^a^	300 ± 28.3 ^b^
Copper (Cu) (mg)	0.9 ± 0.1 ^a^	0.7 ± 0.1 ^a^
Zinc (Zn) (mg)	3 ± 0.3 ^a^	1.7 ± 0.3 ^b^

Values are presented as mean ± standard deviation (SD) of duplicate determinations (*n* = 2). Means in the same row with different superscript letters are significantly different (*p* < 0.05).

**Table 3:** Physicochemical properties of gluten-free cupcakes.

		Oven-Drying	Freeze-Drying	Control
Physical properties	Volume after 24 h (height/weight)	0.07 ± 0.004 ^ab^	0.07 ± 0.003 ^a^	0.09 ±0.008 ^b^
Crumb Color	L*	48.03 ± 0.7 ^a^	53.42 ± 0.9 ^b^	68.25 ± 3.02 ^c^
	a*	9.36 ± 0.7 ^a^	8.45 ± 0.2 ^a^	4.98 ± 0.49 ^b^
	b*	17.64 ± 1.3 ^a^	16.03 ± 0.2 ^a^	29.0 ± 0.88 ^b^
	Brown index (100-L)	51.97 ± 0.68 ^a^	46.58 ± 0.98 ^b^	31.76 ± 3.02 ^c^

Values are expressed as mean ± SD (*n* = 3). L*, brightness; a*, redness-greenness; b*, yellowness-blueness. Means in the same row with different superscript letters are significantly different (*p* < 0.05) according to one-way ANOVA followed by Tukey’s HSD test.

## Data Availability

The original contributions presented in this study are included in the article. Further inquiries can be directed to the corresponding authors.
